# TSE-GAN: strain elastography using generative adversarial network for thyroid disease diagnosis

**DOI:** 10.3389/fbioe.2024.1330713

**Published:** 2024-02-01

**Authors:** Anping Song, Tianyi Li, Xuehai Ding, Mingye Wu, Ren Wang

**Affiliations:** ^1^ School of Computer Engineering and Science, Shanghai University, Shanghai, China; ^2^ Department of Medical Ultrasonics, Shanghai University of Traditional Chinese Medicine Affiliated Shuguang Hospital, Shanghai, China; ^3^ Department of Ultrasound Medicine, Shanghai Sixth People’s Hospital Affiliated to Shanghai Jiao Tong University School of Medicine, Shanghai, China

**Keywords:** deep learning, image translation, generative adversarial networks, strain estimation, ultrasound elastography

## Abstract

Over the past 35 years, studies conducted worldwide have revealed a threefold increase in the incidence of thyroid cancer. Strain elastography is a new imaging technique to identify benign and malignant thyroid nodules due to its sensitivity to tissue stiffness. However, there are certain limitations of this technique, particularly in terms of standardization of the compression process, evaluation of results and several assumptions used in commercial strain elastography modes for the purpose of simplifying imaging analysis. In this work, we propose a novel conditional generative adversarial network (TSE-GAN) for automatically generating thyroid strain elastograms, which adopts a global-to-local architecture to improve the ability of extracting multi-scale features and develops an adaptive deformable U-net structure in the sub-generator to apply effective deformation. Furthermore, we introduce a Lab-based loss function to induce the networks to generate realistic thyroid elastograms that conform to the probability distribution of the target domain. Qualitative and quantitative assessments are conducted on a clinical dataset provided by Shanghai Sixth People’s Hospital. Experimental results demonstrate that thyroid elastograms generated by the proposed TSE-GAN outperform state-of-the-art image translation methods in meeting the needs of clinical diagnostic applications and providing practical value.

## 1 Introduction

Based on high-resolution B-mode ultrasound (US), thyroid nodules have been identified as one of the most prevalent thyroid disorders, with an incidence rate of up to 67% in adults (Samir et al., 2015; Yoon et al., 2015). Differentiating between benign and malignant thyroid nodules is crucial, as the risk of morbidity and mortality increases with the progression of thyroid cancer. Ultrasound elastography (USE) is a noninvasive technique that takes advantage of the changed elasticity or stiffness of soft tissues resulting from specific pathological or physiological processes. For instance, many solid tumors are known to differ mechanically from surrounding healthy tissues. Since thyroid USE can complement B-mode ultrasound and fine needle aspiration (FNA) in assessing thyroid nodules, the combination of thyroid USE with B-mode ultrasound for clinical diagnosis has become increasingly popular, thereby improving the ability to distinguish between benign and malignant thyroid nodules.

**FIGURE 1:**
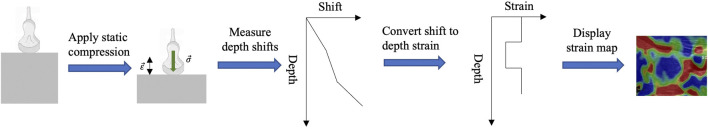
The process and principle of thyroid strain elastography.

Strain Elastography (SE) was the first introduced USE technique. During the inspection, the operator exerts manual compression on the tissue with the ultrasound transducer (Ophir et al., 1991). The SE device is an add-on module to the conventional ultrasound device. When compression is applied in place, both the B-mode ultrasound and the corresponding SE images will be displayed on the screen, which can assist the operator in the stiffness assessment. The process and principle of SE is shown in Figure 1.

However, there are two existing limitations on strain elastography.

Firstly, the process of compressing is difficult to standardize in practice and the stiffness assessment relies heavily on the subjective judgment of operators.

Furthermore, commercially available USE modes rely on a set of assumptions about the tissue material such as linear, elastic, isotropic and incompressible to simplify analysis and interpretation of imaging (Sigrist et al., 2017). However, studies have shown that these assumptions have only held in specific clinical scenarios and are not applicable in other imaging applications. In principle, such assumptions violate conventional models that describe soft tissue mechanical properties as complex and heterogeneous materials that have both a viscous and an elastic mechanical response when probed (Palmeri and Nightingale, 2011).

Recently, the popularity of generative adversarial network (GAN) has greatly promoted the development of generative models and data synthesis techniques, as well as improved the quality and diversity of image generation. Driven by the increasing demand for large datasets and the desire to reduce the cost and time for collecting and labeling, numerous studies have introduced GAN into various medical domains, such as gene design, drug discovery, condition record generation, medical image processing, and elastography.

Elastogram generation can be regarded as an image translation task that transforms an image from a source domain to a target domain. When dealing with thyroid strain elastography, the B-mode ultrasound serves as the source domain, whereas the target domain involves strain elastography ultrasound. Although there are several sophisticated models available for image translation, applying them directly to the elastography task presents certain challenges.

Firstly, the generated images often exhibit misalignment of nodules compared to real images, indicating inadequate feature extraction of crucial regions. In addition, the current models do not adequately consider the probability distribution of SE images in different color spaces, leading to insufficient extraction of color features and inaccurate generation results. Moreover, the existing translation networks lack the ability to accurately estimate the strain at each point during compression, resulting in significant errors in thyroid stiffness assessment.

In order to address the challenges mentioned above, we propose a cGAN-based model called TSE-GAN, which takes into account the nonlinearity, anisotropy, and viscoelasticity of the thyroid during the compression process. TSE-GAN consists of a generator and two discriminators. Specifically, the generator contains three parts: a global generator, a local generator, and a content revisor. The global generator focuses on global image translation; the local generator is designed for strain estimation on thyroid nodules; the content revisor is dedicated to further refining the texture information in both the background and foreground of the generated images. To authenticate the images, we employ two discriminators that evaluate the global and local aspects of the generated images, respectively. Furthermore, through comparing the probability distribution of thyroid SE images in RGB color space and Lab color space, we find that the distribution in Lab space is more concentrated. Therefore, our loss functions are performed in Lab space to impose more effective constraints during the training process. Finally, we evaluate the performance of TSE-GAN against 7 state-of-the-art methods on a clinical dataset. The superiority of the proposed method is supported by several qualitative and quantitative assessments, which are discussed in detail in the experiment section.

The contributions of this paper can be summarized as follows:

• We propose a novel method based on generative adversarial network for thyroid strain elastography, which can transform B-mode ultrasound images to SE images. The network adopts a global-to-local architecture to improve the ability of extracting multi-scale features and develops an adaptive deformable U-net structure in the sub-generator to apply effective deformation on the thyroid.
• We design a new loss function according to the unique probability distribution of thyroid SE images in Lab space, which aims to minimize the difference in color distribution between the source domain images and the target domain images.
• Qualitative and quantitative experiments are conducted on a clinical dataset provided by Shanghai Sixth People’s Hospital, including 1,224 pairs of B-mode ultrasound and SE samples. Results show that the proposed model can generate realistic images with more clear details compared to existing methods.

## 2 Related work

### 2.1 Generative adversarial network

Generative adversarial networks (GANs) are a type of deep learning model composed of two neural networks, namely, the generator and the discriminator. These networks are trained in a game-like framework, where the generator creates synthetic data resembling real data, and the discriminator distinguishes between real and synthetic data. The training process involves an iterative interaction between the generator and discriminator, aiming to improve the quality of generated samples.

Karras et al. (2019) introduced the StyleGAN architecture, which allows for generating images with controllable factors. It enables the separation of different factors like hair, age and gender, facilitating control over the appearance of the generated output. To address GAN’s limitation in capturing consistent geometric or structural patterns in certain categories, SAGAN (Zhang et al., 2019) introduced a self-attention mechanism. Such mechanism enables learning of inter-sequence dependencies and long-range feature relationships on a global scale, resulting in the generation of images with complex geometric constraints. Building upon SAGAN, BigGAN (Brock et al., 2018) was developed as a large-scale implementation. BigGAN incorporates techniques such as increasing batch size, truncation techniques, and controlling model stability, allowing it to generate high-resolution images with detailed backgrounds and rich textures.

### 2.2 Medical image generation

In recent years, the use of GAN networks has been explored extensively in various application scenarios for medical images, including denoising, reconstruction, segmentation, data generation, detection, and classification. For instance, (Bermudez et al., 2018), trained a GAN to synthesize T1-weighted brain MRI images that exhibited comparable quality to real images. (Zhao et al., 2023). proposed a new dual domain Swin Transformer network for MRI reconstruction, which demonstrates a substantial improvement in the network’s feature extraction capabilities, allowing it to effectively capture long-range dependencies in the input data.

GANs are also utilized for generating additional training data. For example, (Painchaud et al., 2020), proposed a combination of variation autoencoder and GAN as a framework for data augmentation in image segmentation tasks. In short, GAN networks are widely used in medical image research, and can generate high-quality and reliable images.

### 2.3 Medical image translation

Medical image translation involves converting medical images from one modality to another, such as MRI to CT or PET to CT. It is a rapidly advancing field in computer vision that aims to enhance the accuracy and efficiency of medical diagnosis and treatment planning. MedGAN (Armanious et al., 2020) introduced a new high-capacity generator architecture that can be applied to various medical tasks without requiring specific modifications for each application. Cycle-MedGAN (Armanious et al., 2019) built upon the widely used CycleGAN framework and incorporated new non-adversarial cycle loss functions. This extension was specifically designed for tasks such as PET to CT translation and MR motion correction. TarGAN (Chen et al., 2021) utilized a novel translation mapping mechanism to improve the quality of the target area during the image generation process. Additionally, it incorporated a shape controller to address the deformation issues caused by untraceable constraints. These features made TarGAN effective in generating whole medical images while alleviating problems related to image deformation.

### 2.4 Ultrasound elastography

Wildeboer et al. (Wildeboer et al., 2020) developed a deep learning model to generate synthetic SWE (sSWE) images from traditional ultrasound images. Their approach utilized a U-Net architecture of a convolutional neural network (CNN) and employed side-view ultrasound and SWE images from 50 prostate cancer patients for research and experimentation. Yao et al. (Yao et al., 2023) proposed a GAN-based model to directly translate ultrasound images into virtual endoscopic ultrasound images. Extensive experiments were conducted to demonstrate good visual consistency and clinical value compared to real endoscopic ultrasound images. It is important to note that these methods primarily focus on shear wave imaging, which differs from strain imaging in terms of imaging principles. Zhang et al. (Zhang et al., 2022) introduced the AUE-Net, which was based on the U-Net architecture and optimized using attention modules and feature residual blocks. However, since their dataset is compression ultrasound images, the raw data acquisition still requires manual compression by operators.

## 3 Methods

According to doctors’ clinical experience, we design an ultrasound translation network called TSE-GAN. The translation network is composed of a generator and two discriminators.

Detailed explanation on the architecture of the generator and discriminator networks are delivered in Section 3.1–3.3 introduces the loss functions employed in the proposed method.

### 3.1 Generator

**FIGURE 2:**
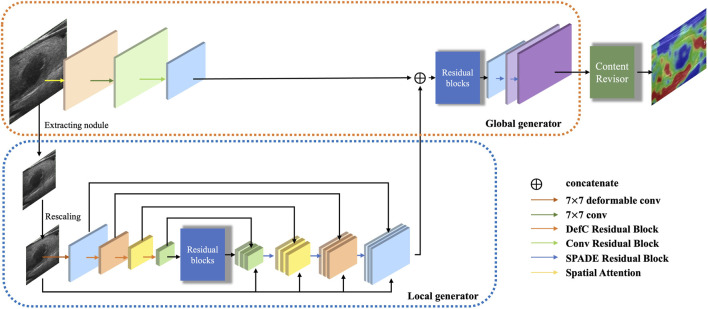
Architecture of the proposed generator.

The generator is responsible for performing transformation on the input B-mode ultrasound image to generate a target SE image. The architecture of the generator *G* is illustrated in Figure 2.

Inspired by Pix2pixHD (Wang et al., 2018), we argue that the multi-resolution pipeline is a well-established practice in computer vision and can effectively aggregate global and local information for the image synthesis task. Therefore, we decompose the generator into three parts: a global generator *G*_*global*_, a local generator *G*_*local*_ and a content revisor *R*. The global generator operates at a resolution of 256 × 256, which targets to deal with the whole B-mode ultrasound image. Then we use a specific preprocessing method to extract the region of interest in size of 128 × 128 (0.5× along each image dimension), as the input of the local generator. Furthermore, we feed the output of the whole generator into a postprocessing module, which we called content revisor, in order to better perceive and generate the most discriminative foreground parts and simultaneously preserve well the unfocused objects and background.

#### 3.1.1 Local generator

Our local generator *G*_*local*_ aims to estimate the implicit strain. *G*_*local*_ consists of three parts: a convolutional encoder, a feature fusion module, and a transposed convolutional decoder.

Given the absence of nodule annotation information in our dataset, we adopt a pre-trained model called BTNet (Li et al., 2023) for thyroid nodule segmentation. This approach allows us to obtain coarse segmentation results, which are then adjusted to a uniform resolution of 128 × 128 as inputs to the local generator.

**FIGURE 3:**
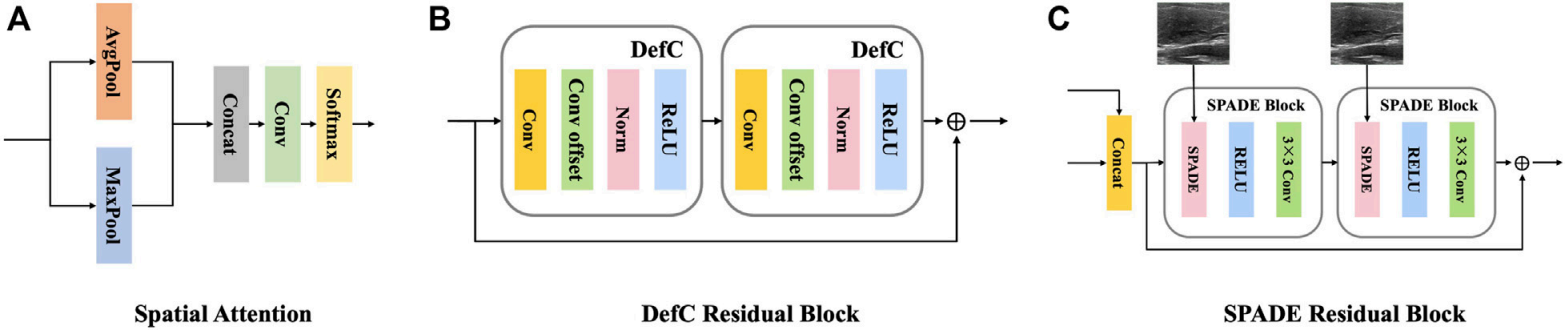
Detailed components of the proposed generator.**(A)** The structure of one spatial attention block. **(B)** The structure of one deformable residual block. **(C)** The structure of one SPADE residual block.

The encoder is composed of a series of deformable convolution residual blocks, which targets to apply geometric transformations to thyroid nodule areas. Each deformable convolution residual block contains two deformable convolution blocks, with a shortcut connection. Each deformable convolution block is composed of four layers: a convolutional layer, a convolutional offset layer, a batch normalization layer, and an activation layer, as shown in Figure 3B.

As is known to all, convolutional neural networks (CNNs) are architecturally invariant to translation, which means the system can exactly produce the same response, regardless of input shifting. However, CNNs are inherently limited to model large, unknown transformations and lack internal mechanisms to handle different geometric transformations, which may cause noticeable problems for non-rigid objects, especially soft tissues like thyroid. The mechanical properties of the thyroid, including nonlinearity, anisotropy and viscoelasticity, imply that different locations may undergo varying scales or deformation during compression. Therefore, adaptive determination of scales or receptive field sizes is desired for precise visual recognition and localization. Considering these reasons, we discard the commonly used convolution blocks in the downsampling stage.

In contrast, deformable convolution (DefC) (Zhu et al., 2019) can significantly enhance CNNs’ capability of modeling geometric transformations by learning offset locations, and thus adaptively decide scales of receptive field with fine localization and achieve the deformation of different scales, shapes and orientations.

For thyroid-related tasks, using large convolutional kernels is more effective for capturing coarse thyroid nodule areas, while small convolutional kernels are better suited for obtaining accurate contour details. Therefore, we further enhance our model by employing multiscale convolutional kernels instead of single-scale kernels. Specifically, we utilize a large convolutional kernel of size 7 × 7 for the first DefC layer and employ small convolutional kernels of size 3 × 3 for subsequent layers. Additionally, the residual design is integrated in the deformable encoder to mitigate the vanishing gradient problem.

The output of the encoder is then fed into nine consecutive residual blocks to achieve underlying feature fusion.

The decoder consists of a succession of SPADE (Park et al., 2019) residual blocks, each of which contains two spade blocks, with a shortcut connection. Each spade block is composed of three layers: a spatially-adaptive denormalization layer, an activation layer and a convolutional layer, as shown in Figure 3C. The input of the spatially-adaptive denormalization layer is combined with skip connection feature maps, upscaled feature maps and raw input images.

**FIGURE 4:**
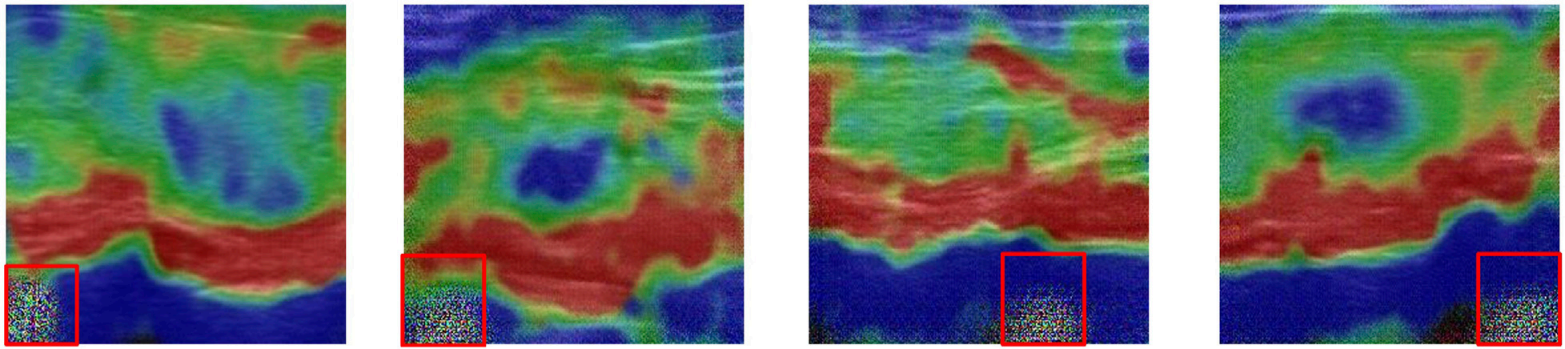
Artifacts that appear near the boundary (highlighted in the red box).

SPADE provides a spatially-variant affine transformation which is learned from the input images for modulating the activation map. Such design can greatly eliminate the boundary artifacts caused by instance normalization which is commonly used in style transfer tasks. Boundary artifacts are shown in Figure 4.

#### 3.1.2 Global generator

Our global generator *G*_*global*_ is designed to transform the ultrasound images in a global perspective. *G*_*global*_ also consists of three components: a convolutional encoder, a set of residual blocks and a transposed convolutional decoder, as shown in Figure 2. The input resolution of *G*_*global*_ is 256 × 256.

Different from *G*_*local*_, the global encoder contains a spatial attention module and a convolutional block. The spatial attention module is used to strengthen the feature extraction weight of the key nodule region in the early stage of the network, so as to more fully extract the features of the nodule and its surrounding areas. The architecture of the spatial attention module is illustrated in Figure 3A.

In addition, the input of the first residual block is the element-wise sum of the output feature map of the global encoder and the output feature map of *G*_*local*_, which helps to integrate multi-scale features given by *G*_*local*_ and *G*_*global*_.

#### 3.1.3 Content revisor

**FIGURE 5:**
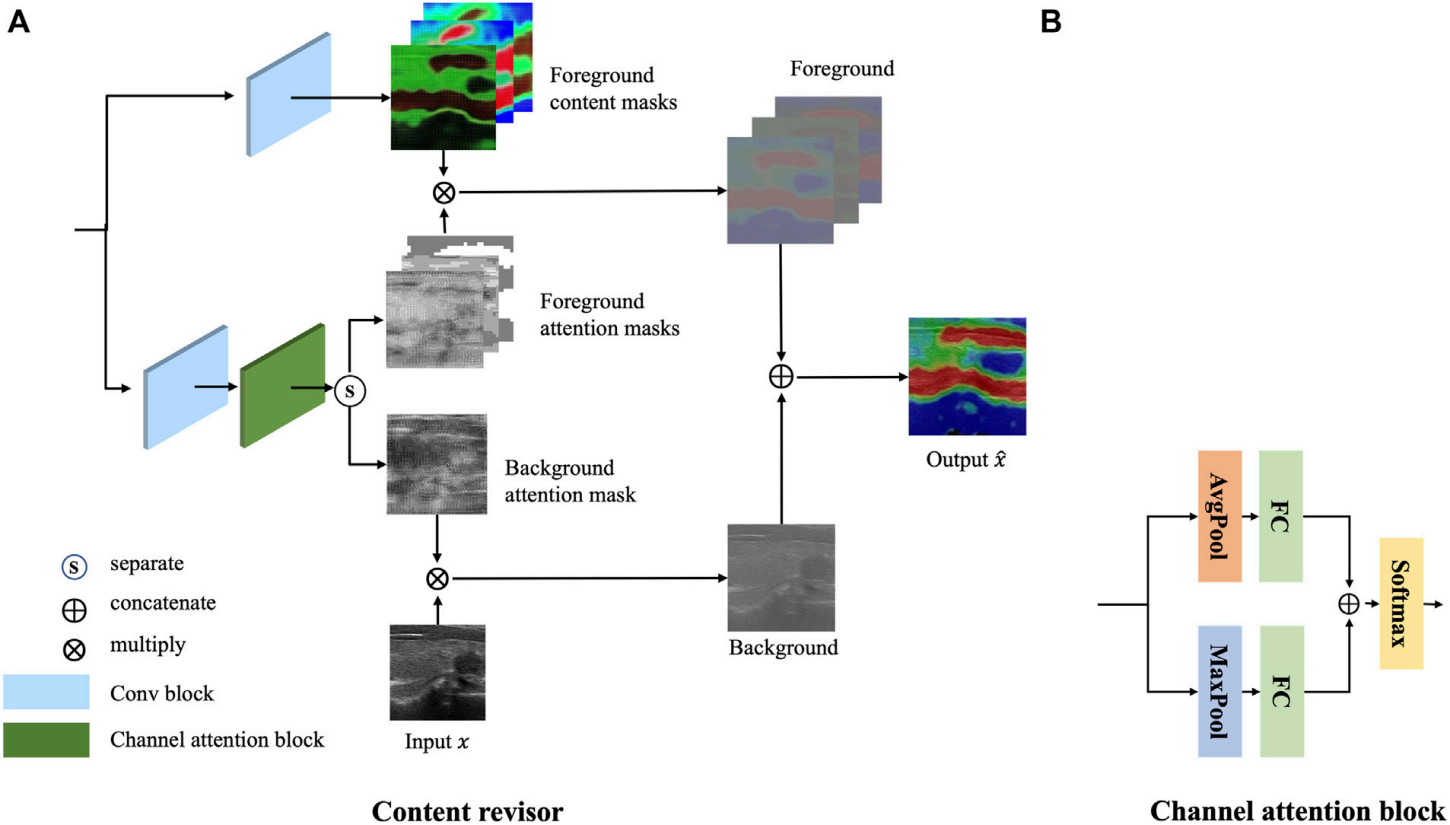
Architecture of the content revisor. **(A)** Content revisor. **(B)** Channel attention block.

Inspired by AttentionGAN (Tang et al., 2021), a novel postprocessing module, called content revisor, is proposed to further modify the foreground of the generated images and simultaneously preserve the background of input images. The content revisor divides the output of the whole generator into *n* groups, where the first *n* − 1 layer is foreground and the last layer is background. Then the content information of each layer is weighted with the corresponding channel attention to get the final output result. In this paper, *n* is set to 4. The architecture of content revisor is shown in Figure 5A.

Specifically, the feature map *m* extracted from *G*_*global*_ is first fed into a convolution block to generate *n* − 1 content masks ${\left\{C_{f}\right\}}_{f=1}^{n-1}$ . The convolution operation is performed with *n* − 1 convolutional filters ${\left\{W_{C}^{f},b_{C}^{f}\right\}}_{f=1}^{n-1}$ . The calculation process of content masks can be expressed as follows:

$$C_{f}=Tanh\left(mW_{C}^{f}+b_{C}^{f}\right),\;\text{ for }\;f=1,\ldots ,n-1$$ (1)

Meanwhile, the feature map *m* is fed into a convolution block and a channel attention module to generate the corresponding attention masks ${\left\{A_{f}\right\}}_{f=1}^{n}$ . The architecture of the channel attention module is shown in Figure 5B. The calculation process of attention masks can be expressed as follows:

$$A_{\mathrm{mid}}=mW_{A}^{f}+b_{A}^{f},\;\text{ for}\;f=1,\ldots ,n$$ (2)

$$A_{f}=Softmax\left(FC\left(AP\left(A_{\mathrm{mid}}\right)\right)+FC\left(MP\left(A_{\mathrm{mid}}\right)\right)\right)$$ (3)

where a convolution operation is performed with several convolutional filters ${\left\{W_{A}^{f},b_{A}^{f}\right\}}_{f=1}^{n}$ . *Softmax*(⋅) is a channel-wise softmax function used for the normalization. *FC*(⋅) represents the full connection layer. *AP*(⋅) and *MP*(⋅) respectively represent average and maximum pooling operations.

We then split ${\left\{A_{f}\right\}}_{f=1}^{n}$ into *n* − 1 foreground attention masks ${\left\{A_{f}\right\}}_{f=1}^{n-1}$ and one background attention mask *A*_*n*_ along the channel dimension.

Finally, the attention masks are multiplied by the corresponding content masks to synthesize the target image *G*(*x*).

$$G\left(x\right)=\overset{n-1}{\underset{i=1}{\sum }}\left(C_{i}*A_{i}\right)+x*A_{n}$$ (4)

where $\sum_{i=1}^{n-1}\left(C_{i}*A_{i}\right)$ represents the foreground part of the generated image, while x*A_*n*_ represents the background one.

### 3.2 Discriminator

For the discriminator in our model, we adopt the PatchGAN(23) framework. We employ two discriminators with the same structure but different parameters to authenticate the outputs of the global generator and the local generator. We will refer to the discriminators as *D*_1_ and *D*_2_, where *D*_1_ stands for the global discriminator and *D*_2_ stands for the local discriminator. The PatchGAN architecture is specifically designed to perform local image-level discrimination, prioritizing the capture of fine-grained details and local structures over global image-level information. It achieves this by dividing the input image into small overlapping patches and applying convolutional operations independently to each patch.

The use of PatchGAN allows our model to effectively capture and preserve intricate details at the patch level, resulting in visually appealing and realistic generated images. By analyzing and processing image patches individually, the model can focus on generating high-quality textures and local variations. This approach not only enhances the overall image quality but also provides greater flexibility in the generation process, enabling the synthesis of diverse and varied images with rich visual details.

### 3.3 Loss function

In this section, the losses of TSE-GAN are discussed. The complete loss is a weighted sum of three losses calculated in CIE Lab color space. Each loss is discussed in detail in the following subsections.

**FIGURE 6:**
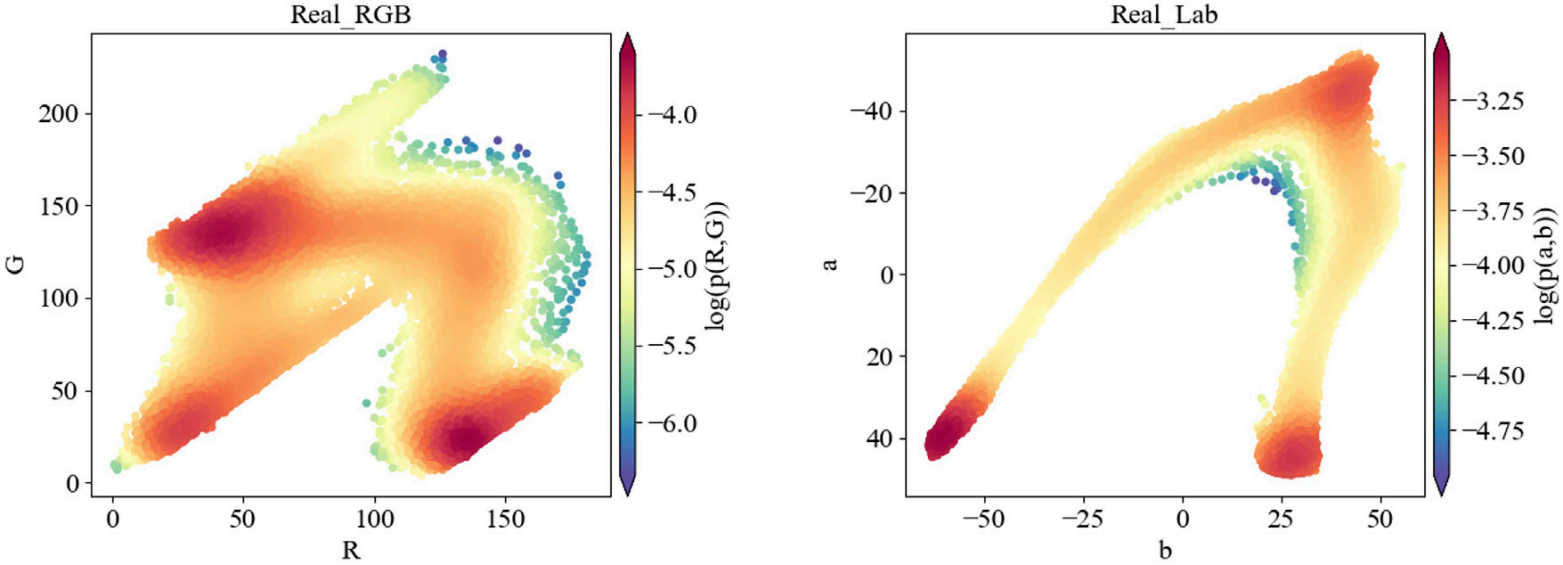
Visualization results of the probability distribution in RGB color space and Lab color space for the same SE image (serial number: 1-389365).

The major colors of thyroid elastic images are red, blue and green, with a few spots showing yellow, while other colors that are common in natural images are hardly noticeable in elastic images. In contrast to RGB space, Lab space is designed based on human’s perception of color, more specifically, it is perceptual uniform. In other words, if the three values L, a, and b are changed by the same amount, the visual variation will be changed by a similar amount. The coordinate axis L represents the luminance, while a and b represent the opposing color dimensions. The larger the L*, the higher the luminance. a* changes from negative to positive, corresponding to a change in color from green to red. b* also changes from negative to positive, corresponding to a change in color from blue to yellow. Therefore, we visualize the probability distribution of SE images in RGB space and Lab space. Results are shown in Figure 6.

As shown in Figure 6, the probability distribution of thyroid SE images in Lab space is more concentrated and the distribution characteristics are more obvious than in RGB space. Therefore, we calculate losses in Lab color space to strengthen the constraints on the training process.

#### 3.3.1 Adversarial loss

The adversarial loss is computed based on the discrepancy between the predicted probability scores of the discriminator for real and generated images. It is formulated using binary cross-entropy loss, where the generator seeks to minimize this loss, while the discriminator aims to maximize it. The adversarial loss is defined as below.

$$L_{\text{GAN}}\left(G,D_{k}\right)=E_{x,y}\left[\log D_{k}\left(x,y\right)\right]+E_{x}\left[\log\left(1-D_{k}\left(x,G\left(x\right)\right)\right)\right]$$ (5)

where *D*_*k*_ stands for sub-discriminators (*D*_1_ for a global discriminator, while *D*_2_ for a local discriminator). *x* stands for the source image and *y* stands for the ground truth image. Such discriminators aim to classify the concatenation of the source image *x* and its corresponding ground truth image *y* as real, written as *D*_*k*_(*x*, *y*) = 1, while classifying *x* and the generated image $\hat{y}$ as fake, written as $D_{k}\left(x,\hat{y}\right)=0$ .

By optimizing the adversarial loss, the generator gradually learns to generate images that closely resemble the real data distribution, leading to the creation of highly realistic and visually appealing images. The adversarial training process helps the generator capture the complex patterns and structures present in the real data, effectively modeling the underlying data distribution.

#### 3.3.2 Feature matching loss

To encourage the generator G to produce outputs $\hat{y}$ that closely resemble the ground truth images *y*, we then incorporate discriminator feature matching loss. This loss promotes training stability by requiring the generator to mimic real image characteristics across various levels. This is achieved by extracting and comparing features from several layers within the discriminator, aiming to align these features from both genuine and generated images. For clarity, the feature extractor at the *i*th layer of discriminator *D*_*k*_ is referred to as $D_{k}^{\left(i\right)}$ . The feature matching loss *L*_*FM*_(*G*, *D*_*k*_) is then calculated as:

$$L_{\text{FM}}\left(G,D_{k}\right)=E_{\left(x,y\right)}\overset{T}{\underset{i=1}{\sum }}\frac{1}{N_{i}}\left[{\left\|D_{k}^{\left(i\right)}\left(x,y\right)-D_{k}^{\left(i\right)}\left(x,G\left(x\right)\right)\right\|}_{1}\right]$$ (6)

where T is the total number of layers and *N*_*i*_ denotes the number of elements in each layer.

#### 3.3.3 Color loss

In order to focus solely on the differences in brightness, contrast, and primary colors of the image while disregarding texture and content, we apply a Gaussian blur to the image. This blurring process helps to eliminate small pixel differences and ensures that the color differences are emphasized. Subsequently, we utilize an additional convolution layer to compute the distance between the feature maps obtained from the blurred images, effectively expressing the color differences between them.

The color loss, denoted as the difference between images *X* and *Y*, is computed based on the above processing steps. This color loss term quantifies the dissimilarity in color distribution between the generated SE image and the real SE image, allowing us to optimize the model towards generating more visually accurate and natural-looking color representations.

In our experiments, we evaluated different distance functions and found that the combination of Euclidean distance and L1 distance yields favorable results. Color loss can be written as:

$$L_{\text{color }}\left(X,Y\right)={\left\|X_{b}-Y_{b}\right\|}_{2}^{2}+{\left\|X_{b}-Y_{b}\right\|}_{1}$$ (7)

where *X*_*b*_ and *Y*_*b*_ are the blurred images of *X* and *Y*, resp.:

$$X_{b}\left(i,j\right)=\underset{k,l}{\sum }X\left(i+k,j+l\right)\cdot G\left(k,l\right)$$ (8)

where *G*(*k*, *l*) denotes the Gaussian kernel with the size of *k* × *l*.

#### 3.3.4 Complete loss

The total loss of our proposed TSE-GAN combined with the adversarial loss, feature matching loss and color loss is as follows:

$$L_{obj}=\underset{k=1,2}{\sum }L_{GAN}\left(G,D_{k}\right)+\alpha \underset{k=1,2}{\sum }L_{FM}\left(G,D_{k}\right)+\beta L_{color}\left(G\right)$$ (9)

where *L*_GAN_(*G*, *D*) is the adversarial loss obtained from Eq. 5. *L*_FM_(*G*, *D*) is the feature matching loss obtained from Eq. 6. *L*_color_(*G*) is the color loss obtained from Eq. 7. *α* is a scalar weight to regulate the importance of *L*_FM_. *β* is a scalar weight to regulate the importance of *L*_color_. By solving the following Eq. 10, the optimal translation model can be obtained.

$$G*=\min_{G}\max_{D_{1}D_{2}}\underset{k=1,2}{\sum }L_{\text{GAN}}\left(G,D_{k}\right)+\alpha \min\underset{k=1,2}{\sum }L_{FM}\left(G,D_{k}\right)+\beta \min L_{\text{color }}\left(G\right)$$ (10)

## 4 Experimental evaluations

### 4.1 Dataset

The dataset used in this study was provided by Shanghai Sixth People’s Hospital, a renowned medical institution in China specializing in the treatment of thyroid disorders. The ultrasound images were obtained from two ultrasound units (both *MylabTwice* ^*TM*^), manufactured by Esaote S.p.A. in Genoa, Italy. A linear array probe (LA523) with a center frequency of 10 MHz was employed for the imaging process.

According to doctors’ experience, B-mode US images and SE images with nodule sizes ranging from 5 mm to 30 mm are valuable for this research, while nodules with coarse calcifications or predominantly cystic characteristics should be excluded. After careful data cleaning, we obtained 1,224 pairs of B-mode US images and SE images from 745 patients spanning the years 2019–2022. Among these patients, there were 213 males and 532 females, with ages ranging from 19 to 84 years. Subsequently, the dataset was randomly divided into a training set comprising 1,129 paired images and a testing set containing 95 paired images.

### 4.2 Training details

All the experiments are implemented using PyTorch and executed on a NVIDIA Tesla P100 with 24-GBVRAM. The network is trained for 1,500 epochs and the batch size is set to 8. The network parameters are initialized using the Xavier method, ensuring a suitable initialization for effective training. To optimize the network, we employ the Adam optimizer with two time-scale update rules, with *β*_1_ set to 0.5 and *β*_2_ set to 0.999. The learning rates for the generator and discriminator are set to 0.0002 and 0.0001, respectively. The loss function hyperparameters *α* and *β* are assigned values of 10 and 0.001, respectively.

In order to improve the robustness and generalization of the model, we incorporate various data augmentation techniques into the training process. These augmentation methods are randomly applied to the input images with a probability of 0.5, including horizontal, vertical, and diagonal translations of ±20 pixels, horizontal and vertical mirror inversions and random rotations within a range of ±15°. Furthermore, to ensure consistent input dimensions for the model, the augmented images are randomly cropped to a size of 256 × 256 pixels.

### 4.3 Evaluation metrics

#### 4.3.1 Graphic index

Due to the lack of consensus in the scientific community regarding the optimal evaluation metrics for assessing the performance of generative models, we employ several traditional image quality metrics, including Peak Signal to Noise Ratio (PSNR), Structural Similarity Index Measure (SSIM), and Mean Squared Error (MSE).

PSNR and SSIM are utilized as indicators of better generation, where higher values signify superior performance. MSE is calculated as the average squared difference between the pixel values of x and y, where lower values signify superior performance. The calculation formulas for these metrics are as follows:

$$\begin{array}{c} PSNR\left(I,G\right)=10\cdot \log_{10}\left(\frac{MAX_{I}^{2}}{\frac{1}{HWC}\sum \|I-G\overset{2}{\underset{2}{\|}}}\right) \end{array}$$ (11)

$$\begin{array}{c} SSIM\left(I,G\right)=\frac{\left(2\mu_{I}\mu_{G}+c_{1}\right)\left(2\sigma_{IG}+c_{2}\right)}{\left(\mu_{I}^{2}+\mu_{G}^{2}+c_{1}\right)\left(\sigma_{I}^{2}+\sigma_{G}^{2}+c_{2}\right)} \end{array}$$ (12)

$$\begin{array}{c} MSE\left(I,G\right)=\frac{1}{HW}\overset{HW}{\underset{i}{\sum }}{\left(I_{i}-G_{i}\right)}^{2} \end{array}$$ (13)

where *I* denotes the real image, *G* expresses the generated image. *H* and *W* are the height and weight of the image. *C* is the number of channels. $MAX_{I}^{2}$ is the maximum pixel value of the image which is 255 here. ${\left\|\cdot \right\|}_{2}$ stands for the Euclidean norm. *μ* and *σ* ^2^ indicate the mean and variance respectively. *σ*_*IG*_ is the covariance. *c*_1_ and *c*_2_ are two variables to stabilize the division with weak denominator.

#### 4.3.2 Elasticity assessment

To quantitatively evaluate the quality of the elastic images generated by TSE-GAN from a clinical perspective, we utilize the Rago criterion as the gold standard for medical assessment.

**FIGURE 7:**
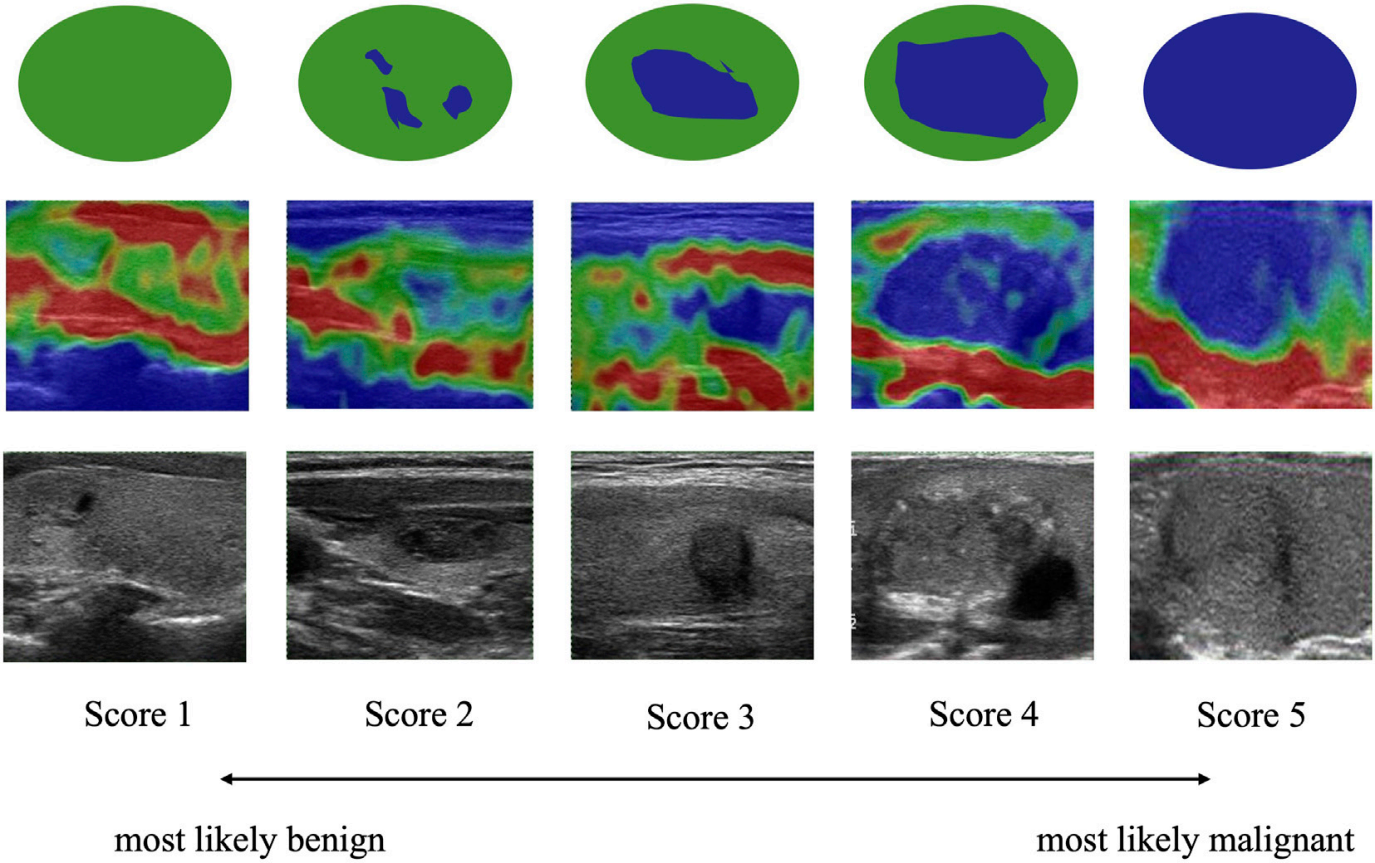
Rago criteria of thyroid nodules. Images of lesions and surrounding tissues showing uniform green color are marked as Score 1. Images that are green overall with a little blue are marked as Score 2. Images with equal blue and green inside the lesion are marked as Score 3. Images that are blue overall with a little green are marked as Score 4. Images of lesions and surrounding tissues showing uniform blue color are marked as Score 5. A higher score indicates a greater likelihood of malignancy.

The Rago criterion, as depicted in Figure 7, employs a scale ranging from 1 to 5 to classify the degree of nodule sclerosis. The score is based on the ratio of blue (indicating sclerotic, inelastic tissue) to green (representing elastic tissue) observed in the elastic images. Score 1 denotes even elasticity in the whole nodule, indicating a higher likelihood of benignity, while Score 5 indicates no elasticity in the nodule, implying a higher probability of malignancy (Rago et al., 2007).

#### 4.3.3 Specialist perceptual study

Finally, we gather all the generated images and present them to the specialists for a visual evaluation of their authenticity and naturalness. These visual evaluations are then combined with the previously mentioned scoring accuracy results to conduct a comprehensive analysis of the performance of the TSE-GAN model.

### 4.4 Results

#### 4.4.1 Ablation study

**FIGURE 8:**
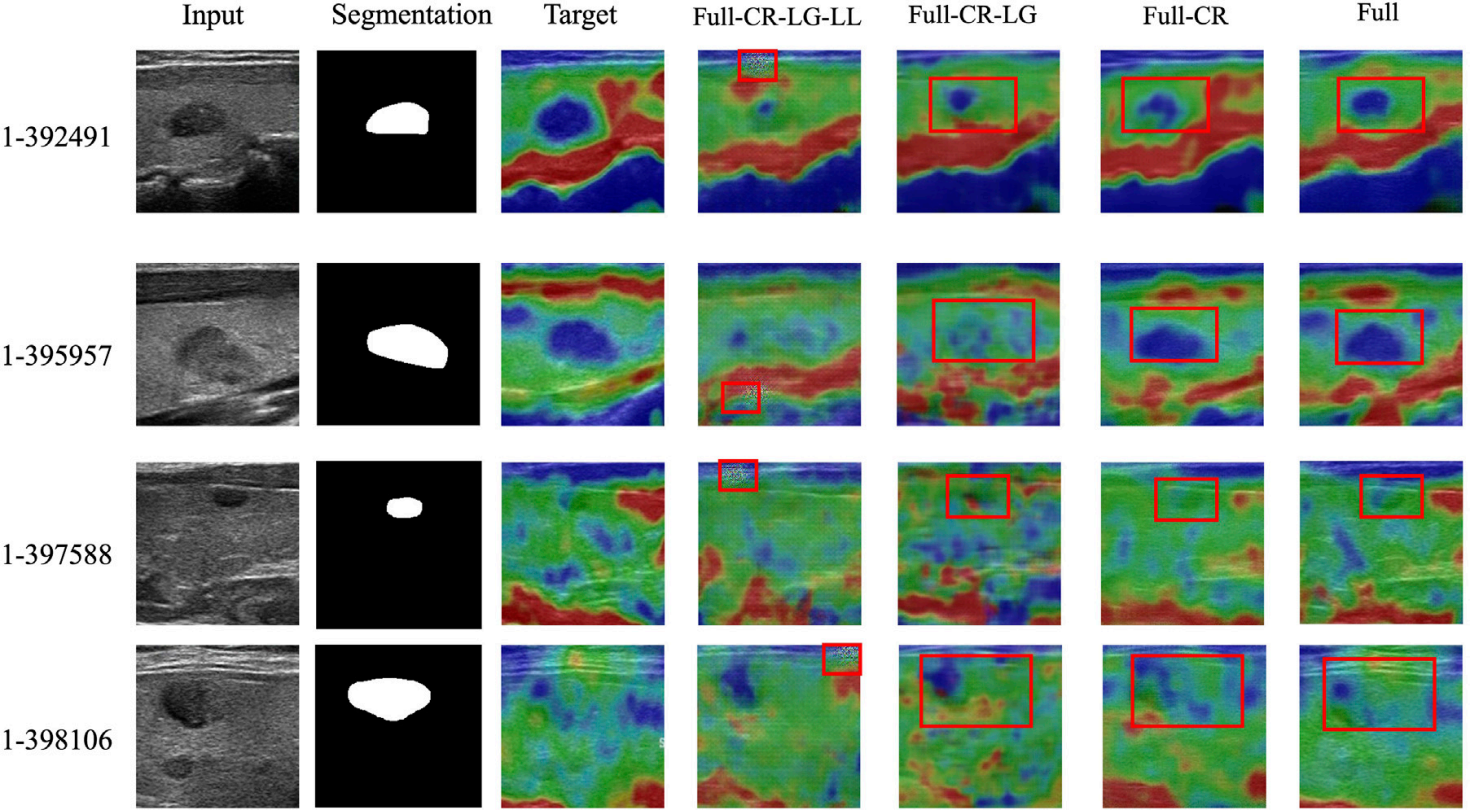
Ablation study of the proposed method. The first column indicates the input ultrasound images. The second column indicates the segmentation masks of the corresponding input images. The third column indicates the real SE images. The rest columns indicate the fake SE images generated by ablation models.

To verify the validity of each module, we conduct an ablation study on the Thyroid Strain Elastography dataset. Table 1 and Figure 8 show the quantitative and qualitative experimental results respectively.

**TABLE 1:** Ablation study of the proposed method. Full stands for proposed model, CR stands for content revisor, LG stands for local generator, LL stands for lab loss. *↑* means that the larger the value of the corresponding objective index, the better the generation effect. Conversely, *↓* means that the smaller the value of the corresponding objective index, the better the generation effect. Bold entries represent the experiments with the best performance.

Methods	PSNR $\left(↑\right)$	SSIM $\left(↑\right)$	MSE $\left(↓\right)$
Full	**28.535**	**0.413**	**91.345**
Full-LG	28.476	0.409	92.514
Full-LG-CR	28.453	0.406	92.991
Full-LG-CR-LL	28.390	0.327	94.300

Note that the nodule segmentation masks displayed in the second column of Figure 8 are purely for indicating the nodule positions in the input ultrasound images. In fact, they are not utilized during the training process. Specifically, our training dataset does not include any segmentation masks.

By systematically removing components of the proposed TSE-GAN, i.e., the Content Revisor (CR), Local Generator (LG), and Lab Loss (LL), we observed a significant degradation in the results. This finding indicates that all of these components are crucial for achieving optimal performance in our approach.

As shown in fourth column of Figure 8, the removal of all three modules resulted in a significant deterioration of the generated images. The edges of the nodules became indistinguishable. Meanwhile, undesired light spots appeared in the images. As shown in fifth column of Figure 8, when CR and LG modules were removed, the blue areas became more prominent, causing blurriness in the nodules in the first row of images. As shown in sixth column of Figure 8, though removing the CR module alone allowed for correct nodule localization, the translation of the blue and green regions remained unsatisfactory. Conversely, with the full model, the extent of nodules’ sclerosis was accurately represented, showcasing the importance of all modules in achieving desirable results.

We also tested the effect of different loss functions on the network training process. In this paper, feature matching loss (*L*_*FM*_) and color loss (*L*_*color*_) are introduced based on adversarial loss (*L*_*GAN*_). To demonstrate the effectiveness of feature matching loss and color loss, networks optimized with different combinations of loss functions, including *L*_*adv*_, *L*_*adv*_ + *L*_*FM*_, *L*_*adv*_ + *L*_*FM*_ + *L*_*color*_, were compared. Quantitative results show that the proposed *L*_*adv*_ + *L*_*FM*_ + *L*_*color*_ objective function significantly outperforms the other comparative loss functions in terms of PSNR, SSIM, and MSE metrics. Specifically, the mean PSNR, SSIM and MSE of the network optimized by *L*_*adv*_ are 28.476, 0.409 and 92.514 respectively. The mean PSNR, SSIM and MSE of the network optimized by *L*_*adv*_ + *L*_*FM*_ are 28.519, 0.411 and 91.836, respectively. The mean PSNR, SSIM and MSE of our loss function are 28.535, 0.413, and 91.345, respectively.

#### 4.4.2 Comparison with state-of-the-art techniques

We choose seven commonly-used image-to-image translation (I2IT) methods. Paired I2IT models include Pix2pix (Isola et al., 2017), Pix2pixHD, LPTN (Liang et al., 2021) and AUE-Net (Zhang et al., 2022), while unpaired I2IT models include CycleGAN (Zhu et al., 2017), AttentionGAN and Qsattn (Hu et al., 2022). We then compare them qualitatively and quantitatively in the Thyroid Strain Elastography dataset.

**Quantitative Comparison.** In this subsection, we compared the performance of TSE-GAN with the aforementioned I2IT methods. The evaluation was conducted using three image similarity metrics and elasticity scores.

Note that in this paper, the elasticity scores of real images and fake images were all given by two experienced ultrasound specialists from Shanghai Sixth People’s Hospital, who possess 12 and 24 years of ultrasound experience, respectively. Meanwhile, to mitigate scoring bias resulting from preconceived notions, we did not disclose the purpose of the study or the image sources to the two specialists prior to scoring. Additionally, the images were presented to the specialists in a random order. After scoring, we calculated the accuracy of each class and the average accuracy over the entire testing set.

The quantitative results of our experiments, focusing on image quality, are presented in Table 2 and Table 3. As shown in Table 2, PSNR, SSIM and MSE of the proposed TSE-GAN are 28.535, 0.413, 91.346, respectively, far exceeding the other seven models. Furthermore, the generated images are given to the doctor for hardness rating according to the Rago criterion. Based on specialists’ opinions, the elastography images generated by our model could meet the needs of clinical diagnostic applications and provide practical value. We reviewed the cases that showed errors from the specialists. As shown in Table 3, the scoring accuracy was 76.5% for Score 1, 70.6% for Score 2, 71.4% for Score 3, 83.3% for Score 4, and 85.7% for Score 5. It is worth noting that the unstable results for Score 4 and Score 5 could be attributed to the limited samples of these two classes. Furthermore, the accuracy for Score 2 and Score 3 is relatively lower than the other classes. This discrepancy may be attributed to the fact that according to the Rago criterion, nodules classified as Score 2 and Score 3 exhibit a combination of significant blue and green areas. Distinguishing between these two classes becomes challenging, leading to a higher likelihood of errors in classification.

**TABLE 2:** PSNR, SSIM, MAE metrics comparison between the proposed model and other translation methods. Bold entries represent the experiments with the best performance.

Methods	PSNR $\left(↑\right)$	SSIM $\left(↑\right)$	MSE $\left(↓\right)$
Pix2pix	28.342	0.385	95.380
Pix2pixHD	28.467	0.395	92.740
LPTN	27.829	0.287	107.203
AUE-Net	28.369	0.313	94.652
CycleGAN	28.369	0.351	94.799
AttentionGAN	28.253	0.348	97.262
Qsattn	28.306	0.352	94.134
TSE-GAN	**28.535**	**0.413**	**91.346**

**TABLE 3:** Elasticity assessment comparison between the proposed model and other translation methods. Second column to sixth column represent individual accuracy for each class. The last column represents the average accuracy across the entire testing set. Bold entries represent the experiments with the best performance.

Methods	Score 1 (%)	Score 2 (%)	Score 3 (%)	Score 4 (%)	Score 5 (%)	Mean_Accuracy (%)
Pix2pix	52.9	52.9	42.9	16.7	28.5	47.37
Pix2pixHD	58.8	61.7	50.0	66.7	42.9	57.89
LPTN	29.4	23.5	28.6	0.0	14.3	24.21
AUE-Net	50.0	44.1	21.4	0.0	14.3	37.89
CycleGAN	44.1	35.3	57.1	66.7	28.6	43.16
AttentionGAN	26.4	29.4	14.3	0.0	14.3	23.16
Qsattn	38.2	35.3	42.9	33.3	71.4	40.00
TSE-GAN	**76.5**	**70.6**	**71.4**	**83.3**	**85.7**	**74.74**

**FIGURE 9:**
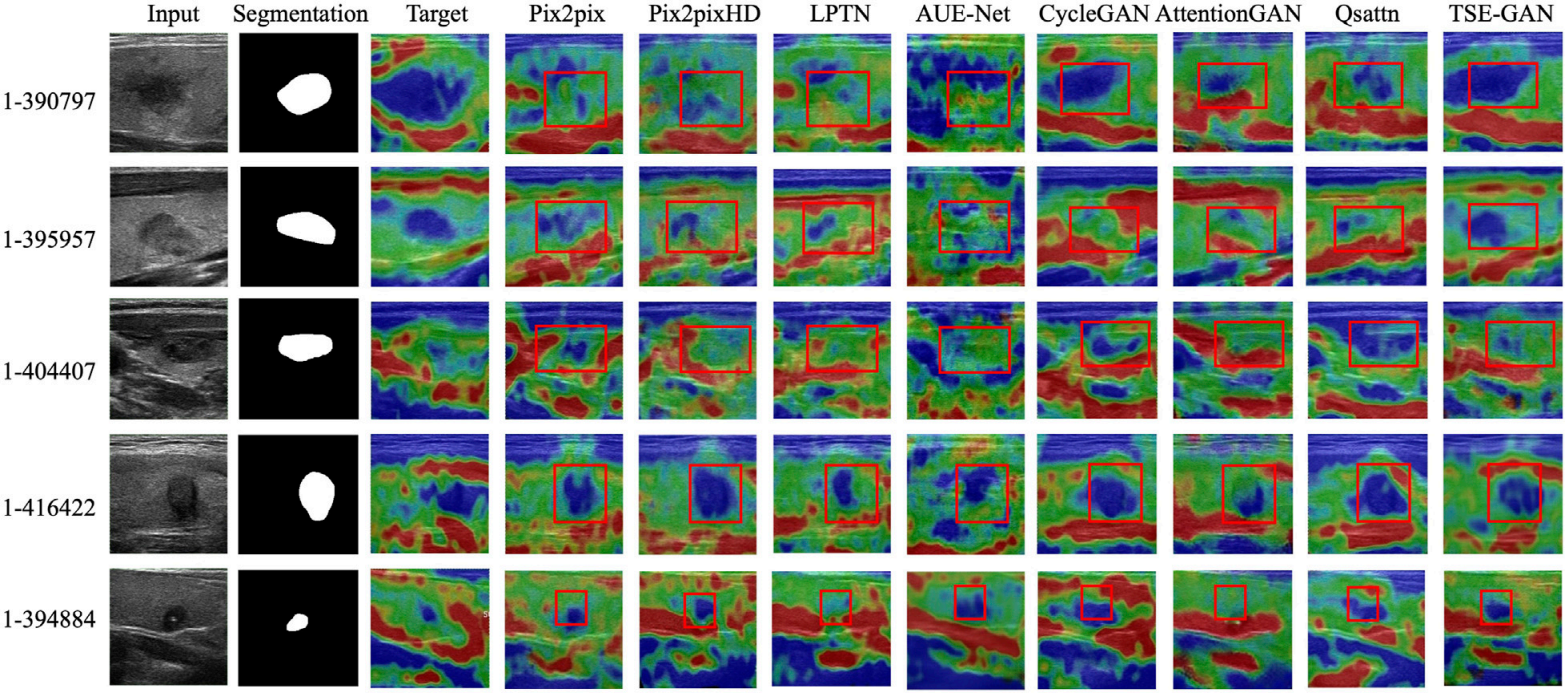
Qualitative results of the TSE-GAN and other I2IT methods.

**Qualitative comparison.** Figure 9 illustrates the qualitative results of the TSE-GAN and other I2IT methods. It is evident that AttentionGAN and LPTN exhibit poor generation effects, as they struggle to accurately locate the nodules. CycleGAN, Qsattn, and Pix2pix tend to label the entire thyroid nodules as Score 5, failing to differentiate between the blue and green regions within the nodules. AUE-Net, on the other hand, excessively emphasizes texture information. Pix2pixHD shows relatively improved results; however, it is worth noting that unwanted changes occur in the background and other objects, as depicted by the red boxes in the first and second rows of Figure 9. In contrast, our proposed method outperforms these existing methods by preserving content details and effectively translating the images into the desired target style.

#### 4.4.3 Specialist perceptual study

In order to evaluate the overall performance of the translation task regarding both the realism and generation effects and to ensure the practical applicability of our method, we invited three medical sonographers from different hospitals to perform a user study based on human perception. In specific, we mixed real and fake SE images and randomly selected 100 images from them. We then presented them to specialists to discriminate whether the image is real or fake. The results are summarized in Table 4.

**TABLE 4:** Accuracy of elasticity assessment from three specialists. Bold entries represent the average accuracy of results given by different doctors.

Specialist	Affiliation	Accuracy (%)
DoctorA(intra-operator, author5)	Shanghai Sixth People’s Hospital	75
DoctorB(intra-operator)	Shanghai Sixth People’s Hospital	81
DoctorC(inter-operator, author4)	Shanghai Shuguang Hospital	73
Mean		**76.3**

Results show that the proposed TSE-GAN achieves a mean accuracy score of 76.3% given by three specialists for the visual performance of realism and generation effect on the ultrasound translation task. The results demonstrate that the proposed method can generate realistic SE images that can confuse the doctors’ judgment.

## 5 Conclusion and discussion

With the development of Strain elastography technique, the combination of SE with B-mode ultrasound for clinical diagnosis has gained popularity because it can greatly improve the distinction between benign and malignant thyroid nodules. In order to eliminate the human element of manual compressing, and break down the assumptions about the tissue material, including linear, elastic, isotropic and incompressible that are commercially available USE modes relied on, we propose a novel method called TSE-GAN, which can generate SE images based on the specific characteristics of thyroid elastography.

The TSE-GAN introduces an adaptive deformable U-net structure with an effective constraint for accurate strain estimation. It also employs a global-to-local architecture to enhance the extraction of multi-scale features, resulting in improved performance. Additionally, a new objective function is designed to minimize the color distribution difference between the source domain and target domain images, taking into account the unique probability distribution of thyroid elastograms in the Lab color space.

However, there are still some limitations in our study. Firstly, TSE-GAN does not translate well for the greater tubercles. Furthermore, since Score 2 and Score 3 are difficult to distinguish, it is necessary to quantify the process of elasticity scoring to reduce the influence of subjective factors. In addition, the training effect of the local generator greatly depends on the results given by the pre-trained segmentation model, which is generally not ideal. Therefore, we will conduct more in-depth research on nodule feature extraction in the future.

## Data Availability

The raw data supporting the conclusion of this article will be made available by the authors, without undue reservation.
